# Daniel Noni Lantum

**DOI:** 10.11604/pamj.2021.38.221.28564

**Published:** 2021-02-26

**Authors:** The Pan African Medical Journal

**Affiliations:** 1 The Pan African Medical Journal, Nairobi, Kenya

**Keywords:** Obituary, iodine-deficiency disorders, Cameroon, Africa, Public health

## Obituary

**Figure 1:**
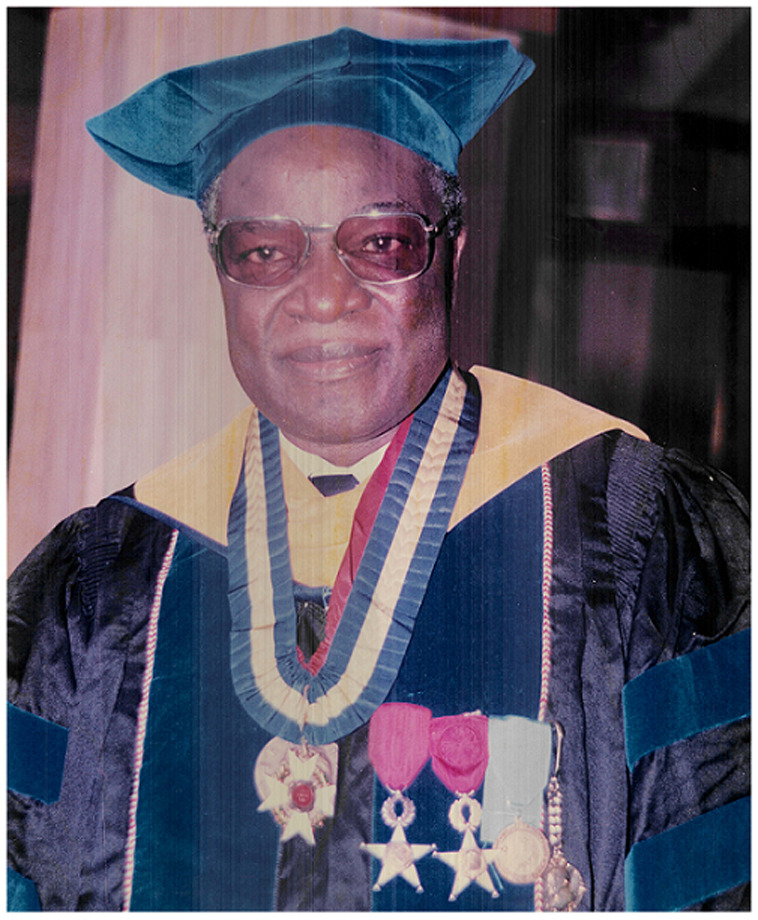
Prof Daniel (Dan) Noni Lantum

Professor Daniel Noni Lantum (Figure 1), an African public health giant is no more. A native of Cameroon, Prof Lantum was born in 1934 in Kumbo, a city in the North West Region of Cameroon and died on 15 February 2021 at the age of 87 years. At the time of his death, he was a Professor Emeritus of the Faculty of Medicine and Biomedical Sciences of the University of Yaoundé I (Cameroon).

He introduced several generations of doctors and public health professionals in his native Cameroon and in Africa to public health practice and directed over 500 theses. Prof Lantum is also well known for his invaluable contribution to the fight against iodine deficiency and associated disorders. His work with the World Health Organization, UNICEF, governments, non-governmental organizations (NGOs) and communities contributed enormously towards universal salt iodization, a key strategy to achieve elimination of iodine-deficiency related disorders for which he was awarded to Algepa Prize in 1996 in Paris and made him well-known in the nutrition and micro-nutrient circles. He is also known to have laid the groundwork for the creation of various faculties of medicine in Africa.

Prof Lantum is also recognized for his passion for African cultural heritage. He worked tirelessly to promote African traditional medicine - that he practiced and taught unapologetically - as an important component of primary health care. Intellectual infatigable, Prof Lantum, wrote intensively in areas as diverse as education reforms, poetry, history, politics, African traditions, and religion.

Prof Lantum studied Medicine at the University of Ibadan (Nigeria), graduating in 1962 with a Bachelor of Medicine and Bachelor of Surgery. He obtained a diploma in tropical medicine and hygiene at the Liverpool School of Tropical Medicine in 1965 and completed a Master (MPH) and Doctorate (DrPH) in Public Health at Tulane University (USA) in 1969 and 1970, respectively. His long and rich professional career encompasses the public sector, NGOs, and development organizations in various African countries. He was a member of numerous professional, traditional and cultural societies.

In his academic career, Prof Lantum was Professor of Public Health at the Yaoundé University Centre for Health Sciences (later known as the Faculty of Medicine and Biomedical Sciences of the University of Yaoundé I) since 1970 and was appointed in 1998 as Vice Chancellor of the Bamenda University of Science and Technology (Cameroon). He received countless distinctions from various governments, organizations, and universities across Africa.

Prof Lantum will be remembered for his passion to transmit knowledge and educate the younger generation, his love for people, and his enthusiasm to address protracted public health issues in Africa. His extraordinary story telling abilities - rooted in his deep knowledge of the traditions and history of his people - translated his passion for African ancestral knowledge. May his soul rest in peace.

He is survived by four children and seven grandchildren.

**Contributions from**: Dr Raoul Kamadjeu (PAMJ, Kenya) and Prof Charles Shey Wiysonge (Cochrane South Africa, South African Medical Research Council, Cape Town, South Africa)

